# Functionalization of a Vegan Mayonnaise with High Value Ingredient Derived from the Agro-Industrial Sector

**DOI:** 10.3390/foods10112684

**Published:** 2021-11-03

**Authors:** Alessandra De Bruno, Rosa Romeo, Antonio Gattuso, Amalia Piscopo, Marco Poiana

**Affiliations:** Department of AGRARIA, University Mediterranea of Reggio Calabria, Vito, 89124 Reggio Calabria, Italy; alessandra.debruno@unirc.it (A.D.B.); rosa.romeo@unirc.it (R.R.); antonio.gattuso@unirc.it (A.G.); mpoiana@unirc.it (M.P.)

**Keywords:** antioxidant activity, mayonnaise, olive mill wastewater, oxidative stability, phenolic extract

## Abstract

This work aimed to evaluate the antioxidant effect determined by the addition of phenolic extract on the oxidative stability and quality of vegan mayonnaise. Two different antioxidant extracts containing 100 mg L^−1^ of hydroxytyrosol and obtained by olive mill wastewater were used in the preparation. After preliminary studies, already evaluated in other works, on hydrophilic and lipophilic food matrices, the results of this study could contribute to understanding the effects of the enrichment on emulsified food systems with phenolic extracts. The functionalized mayonnaise samples were monitored up to 45 days of storage at 10 °C in comparison with a control sample for microbiological, physicochemical, antioxidant, sensory properties and for oxidative stability. The results achieved through this work showed the efficacy of the use of phenolic extract as ingredients for its positive effect on chemical properties of mayonnaise. The adding extracts lead to the increase of oxidative stability with an induction period higher (about 24 h) than the control sample (about 12 h).

## 1. Introduction

Food industries and, particularly, the olive oil industry produce large quantities of by-products that can be a serious environmental problem. Olive mill wastewater could be an economic and natural source of antioxidants due to its high content of phenolic compounds with a wide array of biological activities [1,2]. Scientific researches have shown that their recovery is important at the environmental level for the reduction of pollution; and in food technologies for different aims such as: nutritious, functional agents and for shelf life extension. Afkhami et al. [3] have studied the orange juice enriched with encapsulated polyphenolic extract of lime waste; Romeo et al. [4,5] studied the using the phenolic extract obtained by olive mill wastewater for the enrichment of hydrophilic model system and the application of natural antioxidants in a lipid system (oil).

Mayonnaise represents one of the most widely consumed sauces in the world [6]: it is a semisolid oil-in-water emulsion, prepared traditionally with egg yolk and 60–80% of oil. The presence of egg in mayonnaise formulation is important both for emulsion and for the taste and color but it is a critical point for the health aspect due to the cholesterol amount [7]. Nowadays an increasing number of people is following a vegetarian or flexitarian diet to prevent cardiovascular diseases resulting from bad nutrition. Many scientific works have been carried out on the possibility of the egg’s removal in mayonnaise and replace them with soya, wheat, and milk proteins [8]. For example, soya milk and sunflower oil are used to formulate a vegan mayonnaise. As for all the foods with a high oil content, mayonnaise is susceptible to deterioration due to autooxidation of the unsaturated fats that can negatively affect physicochemical and sensorial attributes of food [9]. Lipid oxidation in mayonnaise causes the development of potentially toxic reaction products, undesirable off-flavors and, simultaneously, it decreases the shelf life of mayonnaise [10,11]. In order to manage these problems, various strategies can be used for avoiding or reduce oxidative processes. One of the common ways to delay lipid oxidation is the use of antioxidants. The efficacy of an antioxidant is influenced by different factors, such as its interaction with other ingredients and its ability to be located at the interface where oxidation takes place.

Generally, synthetic and commercial antioxidants, such as butylated hydroxy toluene (BHT), butylated hydroxy anisole (BHA) and ethylene diamine tetraacetic acid (EDTA), are widely used in mayonnaise to prevent rancidity. However, today the substitution of chemical ingredients with natural ingredients is highly appreciated by the consumer for the health effect and it shows also a great potential for improving food stability against lipid oxidation. Natural antioxidants can act as retarders, when they protect target lipids from oxidation initiators or hinder the propagation phase, the so-called chain-breaking antioxidants [12,13]. For it, the use of plant extracts, rich in antioxidant constituents, such as polyphenols, is an increasing trend in the food industry because they are an alternative to synthetic compounds with reducing and antimicrobial effect [14,15]. The olive oil production generates a considerable amount of olive oil mill waste, rich in organic compounds, mainly phenols. Only a small fraction of phenolic components is transferred to olive oil (1–2%) while the remaining portion is lost in olive oil by-products [16]. This work aimed to evaluate the effect of phenolic extracts obtained by olive mill wastewater in physicochemical and antioxidant characteristics of a vegan mayonnaise during storage period.

## 2. Materials and Methods

### 2.1. Phenolic Extract Preparation

Olive of Ottobratica cv were processed by a three-phase centrifugation apparatus in an olive oil mill located in the province of Reggio Calabria. The obtained olive Mill Wastewater (OMWW) were transferred in Food Technologies laboratory of the Mediterranean University of Reggio Calabria (Italy) where were submitted to two different extraction methods.

Method A*:* was carried out following the method reported by Romeo et al. [4]. An aliquot of OMWW was acidified to pH 2 with HCl and washed three times with hexane (1:1, *v*/*v*) in order to remove the lipid fraction. After shaken and centrifuged (Nüve, Ankara, Turkey) the extraction procedure was carried out by means of ethyl acetate three times and the solvent was recovered in a separating funnel (1:4 *v*/*v*). The ethyl acetate was separated and evaporated using a rotary vacuum evaporator at 25 °C. Finally, the dry residues were again dissolved in 100 mL of water, filtered using PTFE 0.45 μm (diameter 15 mm) syringe filter. The obtained sample, named PE_A_, was then stored at 4 °C until subsequent analyses.

Method B*:* an aliquot of OMWW was acidified to pH 2 with citric acid. After 30 min of shaken and 5 min of centrifugation (6000 rpm) the sample was filtrated with a paper filter and concentrated in an oven at 50 °C. The final residue characterized by gelatinous consistency was extracted with water (1:5, *w*/*v*) in an ultrasound system (Sonoplus Ultrasonic homogenisers, Series 2000.2, HD 2200.2. BANDELIN, Ultraschall seit 1955) for 30 min. Finally, the obtained extract was filtered using PTFE 0.45 μm (diameter 15 mm) syringe filter. The sample, named PE_B_, was then stored at 4 °C until subsequent analyses.

### 2.2. Mayonnaise Preparation

**Figure 1:**
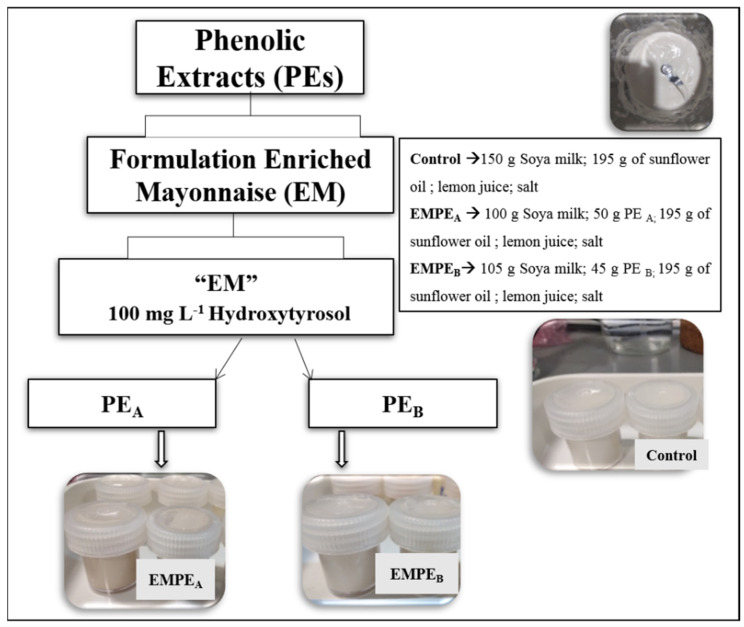
Schematic overview of formulation of mayonnaise.

A schematic overview of the vegan mayonnaise production process is reported in Figure 1. The main ingredients used for the formulation were: soya milk, sunflower oil, lemon juice, salt and phenolic extracts (PE_A_ and PE_B_). All ingredients were mixed using a lab-scale mixer (Bimby TM31, Vorwerk, Wuppertal, Germany) in a three-step process in order to maintain a closely packed emulsion. 1st step: soya milk and salt were mixed (1.100 g·min^−1^, 1 min, 37 °C); 2nd step: sunflower oil and lemon juice was slowly added under continuous mixing (2000 g·min^−1^, 3 min) until a mayonnaise emulsion had been formed; 3rd step: the PE_A_ and PE_B_ amounts corresponding to 100 mg L^−1^ of Hydroxytyrosol (respectively, 50 and 45 g) were incorporating to the mixture (300 g·min^−1^, 1 min). Mayonnaise samples were stored in capped containers and refrigerated at 10 °C for storage. All analyses were performed at 0, 15, 30 and 45 days of storage. The two enriched mayonnaises, EMPE_A_ and EMPE_B_, were compared with a sample without PE, named control.

### 2.3. Antioxidant Characterization of Phenolic Extracts

The main antioxidant parameters, such as: total phenol content (TPC), ABTS and DPPH assays, were performed spectrophotometrically following the method described by De Bruno et al. [16], with some modifications.

For TPC analysis, 0.1 mL of the phenolic extracts (PE_A_ and PE_B_), were placed in a 25 mL volumetric flask and mixed with 20 mL of deionized water and 0.625 mL of the Folin Ciocalteau reagent. After 3 min, 2.5 mL of a saturated solution of Na_2_CO_3_ (20%) were added. The content was mixed and diluted to volume with deionized water. Thereafter, the mixture was incubated for 12 h at room temperature and in the dark. The absorbance of the samples was measured at 725 nm against a blank using a double-beam ultraviolet-visible spectrophotometer (Agilent 8453 UV–Vis, Germany) and compared with a gallic acid calibration curve (concentration between 1 and 10 mg L^−1^). The results were expressed as mg of GAE 100 mL^−1^.

For DPPH assay, 10 µL of PE extracts (PE_A_ and PE_B_) were added to 2990 µL of a 6 × 10^−5^ M of methanol solution of DPPH (2.2-diphenyl-1-picrylhydrazyl, Carlo Erba, MI, Italy) in a cuvette and left in the dark for 30 min (till stabilization). The decrement of absorbance was determined by a spectrophotometer at 515 nm against methanol as blank and at the temperature of 20 °C.

For ABTS assay, 10 µL of PE extracts (PE_A_ and PE_B_) were added to 2990 µL of ABTS reaction mixture (2.20-azino-bis (3-ethylbenzothiazoline-6-sulfonic acid), and the absorbance was measured after 6 min at 734 nm against ethanol as blank by a spectrophotometer. For both the assays, the radical scavenging activity was plotted against Trolox concentration (from 1.5 to 24 µM) and the results were expressed as µmol Trolox mL^−1^ of PE.

The quantification of the main phenolic compounds was carried out following the method described by Romeo et al. [4], through a UHPLC-DAD analysis. 5 µL of PE was injected in a UHPLC system that consisted of an UHPLC PLATINblue (Knauer, Berlin, Germany) equipped with a binary pump system using a Knauer blue orchid column C18 (1.8 µm, 100 × 2 mm) coupled with a PDA-1 (Photo Diode Array Detector) PLATINblue (Knauer, Berlin, Germany). The mobile phases were (A) water acidified with acetic acid (pH 3.10) and (B) acetonitrile; the gradient elution program consisted of 0–3 min, 95% A; 3–15 min, 95–60% A; 15–15.5 min, 60–0% A. Finally, returning to the initial conditions was achieved during analysis keeping the column at 30 °C. External standards (concentration between 1 and 100 mg L^−1^) were used for the quantification and the results were expressed as mg 100 mL^−1^.

### 2.4. Physicochemical, Microbiological and Antioxidant Evaluation of Enriched Mayonnaise Samples (EM)

#### 2.4.1. Physicochemical Analysis

The pH of mayonnaise samples was measured at 25 °C using a digital pH meter (Crison Basic 20, Spain) according to AOAC [17]. Total acidity (% oleic acid) was performed according to Official and standard methods (AOCS, [18,19,20]). The moisture content (MC, %) was tested in an Electronic Moisture Analyser MA37 (Sartorius, Goettingen, Germany). The analysis was performed using 5 g of the sample at 105 °C. The color analysis was evaluated using a reflection colorimeter (Minolta CR 300, Japan) with reference to a CIE L*a*b* coordinates by using a D65 illuminant. Each sample was homogeneously distributed into a glass vessel and the color was recorded at 10 different points.

#### 2.4.2. Microbiological Analysis

The viable populations of the principal groups of microorganisms were determined by plate inoculation and incubation at 32 °C up to 3 days before counting the colonies in the following selective media: total mesophilic bacteria in Plate Count Agar (Plate Count Agar, Conda-Pronadisa, Spain), lactic acid bacteria in MRS Agar (LAB) (Oxoid), yeasts and moulds in OGYA (Oxoid).

#### 2.4.3. Oxidative Stability in Accelerated Storage Test

To investigate the effect of PE extracts in delaying or inhibiting of fat oxidation, mayonnaise samples with and without extract were subjected to high oxidative stress in OXITEST reactor. Oxitest analysis allows to detect the time necessary to reach an end point of oxidation that corresponds to a detectable rancidity or a rapid change in the oxidation rate. An oxidation Test Reactor (VELP Scientifica, Usmate Velate, MB, Italy) was used in order to evaluate the opposition to fat oxidation. This method is recognized by AOCS International Standard Procedure (Cd 12c–16) for the determination of oxidation stability of food, fats, and oils (AOAC, [21]). The analysis consists of monitoring the oxygen uptake of the reactive constituent of food samples to determine the oxidative stability under conditions of accelerated oxidation. Briefly, 5 g of oil sample were distributed homogenously in a hermetically sealed titanium chamber; oxygen was purged into the chamber up to a pressure of 6 bar. The reactor temperature was set at 90 °C. These reaction working conditions allow obtaining the sample Induction Period (IP) within a short time. The OXITEST allows to measure the modification of absolute pressure inside the two chambers and, through the OXISoftTM Software (Version 10002948 Usmate Velate, MB, Italy), automatically generates the IP expressed as hours by the graphical method.

#### 2.4.4. Analysis of Antioxidant Compounds

The extraction of antioxidant compounds from Mayonnaise samples (EM) and the evaluation of antioxidant parameters were carried out following the method reported by Romeo et al. [5], opportunely modified. Two grams of EM were added with 2 mL of methanol: water (70:30) and 2 mL of hexane and mixed with a Vortex for 10 min. The hydro-alcoholic phase was separated from the oil phase in a refrigerated centrifuge apparatus (NF 1200R, Nüve, Ankara, Turkey) at 5000 rpm, 4 °C for 10 min. Hydro-alcoholic extracts were recovered with a syringe, filtered through a 0.45 µm nylon filter, diameter 15 mm (Thermo Fischer Scientific, Waltham, MA, USA), and utilized for the phenolic compounds quantification and antioxidant activity.

For the total phenolic determination in EM, an aliquot of the diluted extract was mixed with 0.300 mL of Folin reagent and 0.25 mL of deionized water and, after 4 min, with 2.4 mL of an aqueous solution of Na_2_CO_3_ (5%). The mixture was maintained in a 40 °C water bath for 20 min and TPC was determined at 750 nm. The results were expressed as mg of gallic acid equivalent kg^−1^ of Mayonnaise. The total antioxidant capacity assays (DPPH and ABTS) and the determination of the main bioactive phenolic compounds in EM samples were analysed with the same methods reported in Section 2.3, with some modifications. For DPPH and ABTS assays, the radical scavenging activity was expressed as µmol Trolox 100 g^−1^ of EM; while the individual phenolic compounds were expressed as mg kg^−1^ EM.

#### 2.4.5. Sensory Evaluation

Sensory characteristics including colour, flavour “taste and odour”, consistency, appearance, overall acceptability was evaluated in EM. The test was performed by a panel of 8 judges (males and females) from 25 to 50 years old, recruited among researchers and technicians of the Food Science and Technology Unit of Reggio Calabria University with previous experience in sensory analysis. The judges were trained before the sessions to identify the attributes to be evaluated Sensory data were elaborated by calculating the median of results.

### 2.5. Statistical Analysis

Results of the present study were expressed as mean ± SD of three measurements (*n* = 3). Appropriate test statistics, Multivariate and One-way ANOVA with Tukey’s post-hoc test, and t-test were at *p* < 0.05 were performed by SPSS Software (Version 15.0, SPSS Inc., Chicago, IL, USA).

## 3. Results

### 3.1. Characterization of Phenolic Extracts

The main antioxidant parameters evaluated on the two phenolic extracts (PE_A_ and PE_B_) were reported in Table 1. Significant differences were noted between the extracts, particularly for TPC and ABTS assays with higher results in PE_A_ (TPC: 7895 mg GAE L^−1^ PE; ABTS: 28,604 µmol TE mL^−1^ PE) respect to PE_B_ (TPC: 7258 mg GAE L^−1^ PE; ABTS: 25,716 µmol TE mL^−1^ PE).

Considering the partition coefficient of wastewaters phenols mixture, the extraction with ethyl acetate by different steps allows retaining most phenolic compounds soluble in the organic phase, as reported by Soberón et al. [22]. This explains the results observed in PE_A_. On the other hand, PE_B_ was obtained by water extraction, so it was characterized by phenols insoluble in the organic phase. The only problem linked to the extract PE_A_ could be represented by the typology of solvents used for the extraction, namely hexane and ethyl acetate, but the extractive procedure was carefully carried out with the aim to use the obtained antioxidant as functional ingredients in food matrixes. For this reason, the solvent has been totally evaporated at the end of the extraction and the solutes had to be recovered with water. To verify if traces of solvent (hexane and Ethyl acetate) persisted in the phenolic extract before the food application, we analyzed the headspace of the hydrophilic phase (through a GC-MS) and have proved absent.

Similarly, differences in antioxidant activity of the phenolic extracts may be ascribed to the polarity of extracting solvents and thus to the chemical characteristics of extracted compounds [23]. The antioxidant activity measured by ABTS assay showed a higher value for both extracts compared to DPPH assay. Likewise, Bibi Sadeer et al. [24] investigated that ABTS cationic radical showed high solubility in organic and aqueous media, thus it is capable to screen the activity of both lipophilic and hydrophilic compounds. In contrast, DPPH radical dissolves in an organic medium reacting only with lipophilic phenolics. The principal phenolics in the extracts were hydroxytyrosol (PE_A_: 759 and PE_B_: 837 mg 100 mL^−1^) and tyrosol (PE_A_: 152 and PE_B_: 148 mg 100 mL^−1^), in agreement with literature [5,25]. Di Mattia et al. [26] reported that tyrosol and hydroxytyrosol are effective in preventing primary and secondary oxidation in o/w emulsion ensuring the oxidative stability during storage.

### 3.2. Qualitative and Quantitative Characterization of Enriched Vegan Mayonnaise (EM)

#### 3.2.1. Physicochemical Aspects

The colour of enriched mayonnaise was evaluated after the phenolic enrichment, considering that it is the main parameters which affect the consumer’s choice (Table 2).

Moreover, the monitoring of its colour was considered crucial to verify the formation of compounds following an oxidative deterioration. The replacement of ingredients compared with the traditional formulation of mayonnaise, leads to a physical and chemical variation, and can have an effect on colour of the final products [27], in particular in this study which involved the use of brown extracts. The addition of phenolic extracts (PEs) and the storage time promoted a significant variation of colour parameters (*p* < 0.05) of enriched mayonnaise. Lightness decreased after phenolic extracts (PEs) addition, more with PE_B_, whereas vegan mayonnaises denoted higher a* and b* parameters after the enrichment. Storage time leads an increase of yellowness parameter and a decrease of redness parameter showing a trend opposite to that proved by Altunkaya et al. [28]. In contrast, no variations were observed for redness between the first and the last day of storage for Control sample. Previous research has proved that colour parameters, in particular lightness, are related to fat droplet sizes [29]. Probably, the modification of fat droplet size that occurred following the addition of phenolic compounds may produce the colour detected changes [30].

**Figure 2:**
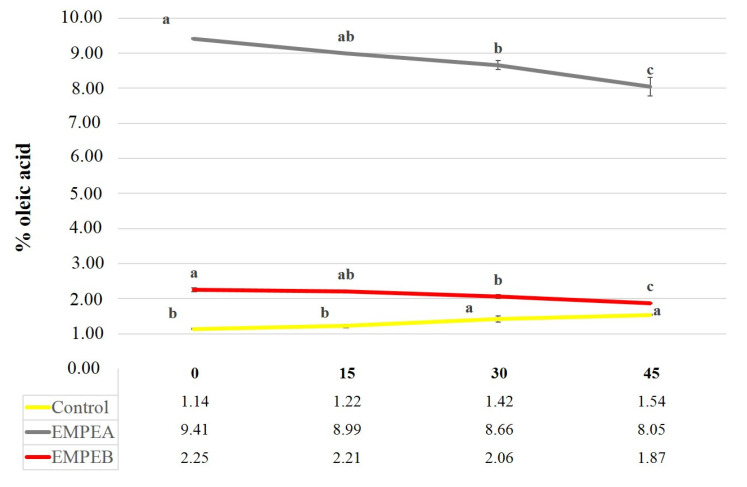
Changes in Total Acidity in the samples during storage time. Different letters show differences for *p* < 0.05.

Food safety and quality are important to consumers. As it is well known, the pH, acidity values and moisture content play an important role in chemical and microbiological stability of fat foods. For this, in order to evaluate the potential application of PEs, all samples were subjects to chemical and microbiological analysis. The pH values of mayonnaise samples analysed at 1st time ranged from 2.92 to 5.01 (Control: 5.01 > EMPE_A_: 2.92 > EMPE_B_: 3.74), therefore, the addition of PE allows an acidification of the enriched samples. slightly lower pH value (Control: 4.97 > EMPE_A_: 2.97 > EMPE_B_: 3.85) were observed at the end of storage period (45 days) according to Rasmy et al. [31]. The two enriched samples showed a decrease of the TA during the time of storage (Figure 2), instead the control sample showed increase of acidity. The highest measured value was in the sample EMPE_A_ (9.41 g Oleic acid 100 g^−1^ mayonnaise). The high acidity value is consistent with pH of PE (pH 2), that induced a decrease of emulsion pH and an increase of TA value.

**Figure 3:**
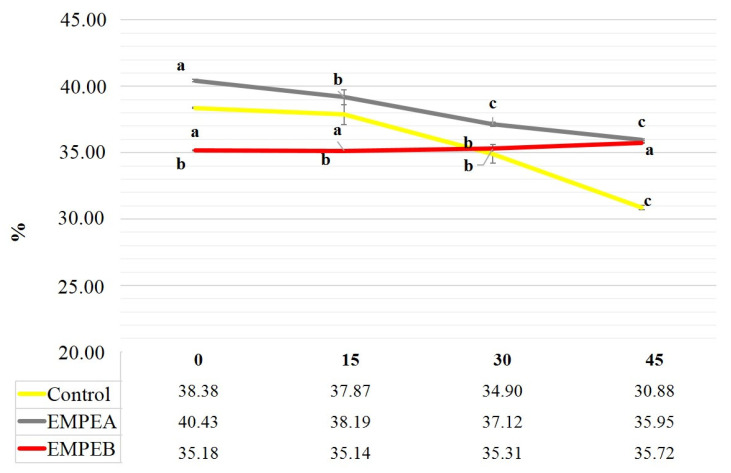
Changes in Moisture content in the samples during storage time. Different letters show differences for *p* < 0.05.

The highest moisture content value was determined in EMPE_A_ sample (40.43%) at 1st time, this value decreased during the storage period, indeed at the 45th day was of about 35.95% (Figure 3). In addition, the Control sample showed a significant variation of the moisture content from 38.38 to 30.88%. EMPE_B_ showed instead a slight, no significant variation over time (35.18 to 35.72%).

#### 3.2.2. Microbiological Parameters

All samples were evaluated for mesophilic aerobic, yeast, moulds and lactic bacteria count. The results detected in all samples were below quantification limits (<1 cfu/mL, data not shown) for 45 days, accordance with that reported also by Martillanes et al. [29]. Probably, the pH conditions of the mayonnaise samples prevented the growth of food spoilage microorganisms [10], acting as an antimicrobial agent.

#### 3.2.3. Sensory Parameters

**Figure 4:**
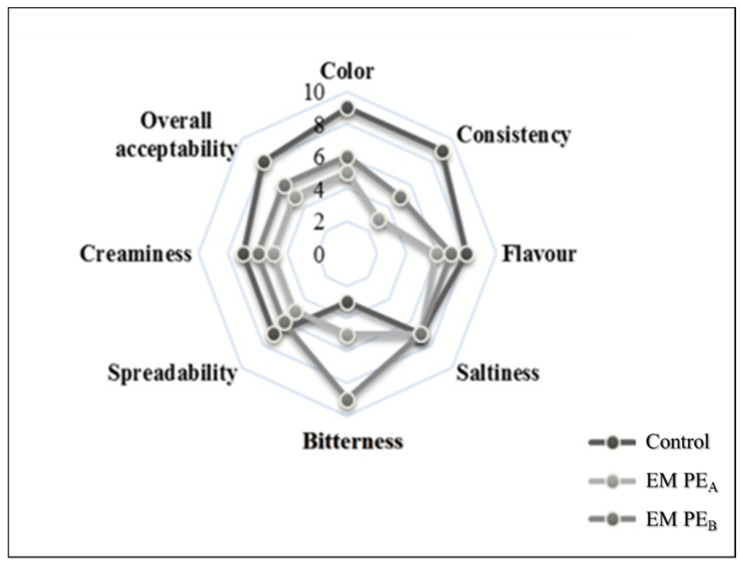
Spider plot of sensory attributes of mayonnaise samples.

The enrichment with PE, leads to a variation of sensory parameters compared to the control sample (Figure 4). In general, all the tested samples showed differences for descriptors among them, except for saltness. Flavour, bitterness, spreadability were affected by the addition of PE; in particular, the increase of the perception of bitterness meant such as acid and pungent taste, was linked probably to the high acidity of the PE_B_ extract (pH 2), as well as to the amount of oleuropein occurred in the enriched samples, acknowledged as responsible of bitter tasting. The natural proteins present in soya milk, determine the formation of the emulsion. Enriched Mayonnaise resulted less consistent than the control sample, probably due to the partial substitution of soya milk with PE. As described by Giacintucci et al. [32], the incorporation of PE in fact modifies the dispersion degree of emulsion with consequences on hardness, consistency end elasticity of samples. Overall, although the sensory evaluation reveals that the addition of PE interferes with the main sensorial attributes, the overall acceptability of EMPE_B_ can be considered good compared to the control sample.

#### 3.2.4. Oxidative Stability and Antioxidant Activity of EM

**Figure 5:**
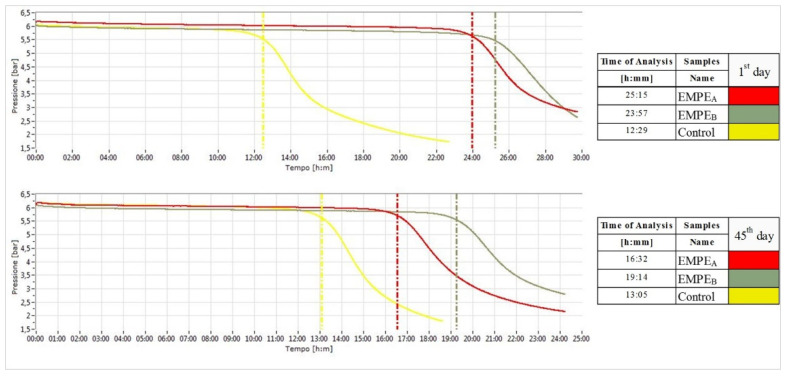
Oxidation curves at 1st and 45th day of storage: red (EMPE_A_), dark grey (EMPE_B_), yellow (Control).

The rate of lipid oxidation in an emulsion is influenced by several factors, including the molecular structure of lipids, heat, light, physical characteristics of emulsion droplets and processing conditions [33]. As it can see in Figure 5, at 1st day of production, enriched samples showed the longer induction period (EMPE_A_: 25:15 h and EMPE_B_: 23:57 h) compared to the control sample (13:05 h). Although, after 45 days of storage lower induction periods were observed in all samples, PEs seems to exert a protective role on thermal oxidative stability of emulsions. At the end of storage, the resistance to rancidity was found to be of 33% and 58% higher rather than the Control for EMPE_A_ and EMPE_B_, respectively.

Likewise, Raikos, [34], showed that the addition of natural antioxidant can be a reliable strategy to improve the resistance to lipid oxidation of fat emulsion. In a previous article written by Paradiso et al. [35], it was reported that the catechol structure characterizing, e.g., hydroxytyrosol and oleuropein exerts a marked inhibiting activity towards oxidation in emulsion. To verify the actual formulation effect on the inhibition of rancidity processes, chromatographic quantification and antioxidant evaluation were performed. UHPLC analysis showed that the main compounds were transferred from extracts to emulsion. The highest content of hydroxytyrosol was detected in EMPE_A_ (82.75 mg kg^−1^) while similar content of tyrosol was quantified in the two EMPE (EMPE_A_ 19.28 and EMPE_B_ 18.12 ± 0.16 mg kg^−1^). Even though, after 45 days, a significant decrease of Hydroxytyrosol was detected equal to 46% for EMPEA sample and 41% for EMPE_B_. It is conceivable that the concentration of bioactive compounds was still relevant in term of antioxidant efficiency. However, it is important to point out that mayonnaise is a multiphase system. In this regard, the polarity antioxidant of different compounds which in turn affects their partition into the different phases, play a key role in antioxidant real effectiveness [36].

In view of the above, multiple assays, TPC, DPPH, and ABTS were performed to allow a full insight into the antioxidant capacity of extracts. As reported in Table 3, the scavenging effect of PE_B_ extracts against ABTS radical cation showed the same trend of TPC. Either way, no significant variations were observed during storage (*p* > 0.05). Only a decrease of 9% in ABTS^+^ results were observed for samples enriched with PE_A_ extract. The addition of PE_B_ extract had a radical scavenging potential against DPPH radical: 134 µmol TE 100 g^−1^ after 1st day while. 78 µmol TE 100 g^−1^ were instead measured in MPE_A_ at the same storage time. Nevertheless, ANOVA data elaboration reveals a significant effect of storage time on EMPE_B_. After 45 days a decrement of 25% of antioxidant activity was detected for EMPE_B_ while no significant variation was observed for EMPE_A_. The highest results of antioxidant activity were showed by ABTS assay, particularly in EMPE_B_ samples with values greater than 610 µmol TE 100 g^−1^. The results obtained from different assays can be correlated to the polarity of compounds present in the food matrix (hydrophilic or lipophilic) for this reason, the antioxidant efficiency has responded better with ABTS test.

## 4. Conclusions

Based on the results, the use of different extracts is a valuable choice to improve the qualitative characteristics of O/W emulsions. The specific phenolic composition of extracts plays a key role in the nutritional parameters of vegan mayonnaise. The concentration of hydroxytyrosol and tyrosol transferred in the samples allows slowing down undesirable oxidation process improving the shelf life of products. In addition, the parameters related to the antioxidant capacity of extracts, TPC, DPPH and ABTS assays, evidenced that the enrichment could have potential health—properties for consumers. Even so, all antioxidant assays indicated that the phenolic extracts had high antioxidant activity and for this reason could be considered suitable for use as a high value−added ingredient.

Finally, the results obtained with the use of the phenolic extract (PE_B_), recovered with the use of only water as a solvent for mayonnaise formulation, opens the way to green methodologies in the recovery of added value molecules from wastewaters. An improvement of the recipe for vegan mayonnaise preparation, with the aim to increase the acceptability of the consumer, could be acquired by adding some ingredients that allow improving the color and taste at an aromatic level.

## Figures and Tables

**Table 1:** Antioxidant parameters and individual Phenolic Compounds of phenolic extracts.

Antioxidant Properties	PE_A_	PE_B_	Sign
***DPPH***	1156 ± 18	1071 ± 9	*
***ABTS***	25,716 ± 35	28,604 ± 18	**
***TPC***	7895 ± 8	7258 ± 14	**
***Hydroxytyrosol***	759 ± 1	837 ± 4	**
***Tyrosol***	152 ± 2	148 ± 0.3	**ns**
***Chlorogenic Acid***	17 ± 0.1	16 ± 0.3	**ns**
***Vanillic Acid***	39 ± 0.0	40 ± 0.5	**ns**
***Caffeic Acid***	26 ± 0.2	21 ± 0.3	**
***p-coumaric Acid***	64 ± 0.3	61 ± 0.0	**
***Oleuropein***	28 ± 0.8	75 ± 0.3	**

Note: The data are presented as means ± SD. Student’s t test performed between the two phenolic extracts (PE_A_ and PE_B_): * significant difference at *p* < 0.05; ** significant difference at *p* < 0.01. ns not significant. µmol TE mL^−1^ PE for ABTS and DPPH and mg 100 mL^−^^1^ PE for TPC and single phenolics.

**Table 2:** Colour parameters of mayonnaise during storage period (days).

Parameters	Time	Control	EMPE_A_	EMPE_B_	Sign.
L*	**0**	89.03 ± 0.39 ^aA^	82.10 ± 0.47 ^bB^	76.26 ± 0.20 ^cB^	**
	**15**	88.93 ± 0.54 ^aAB^	82.33 ± 0.33 ^bB^	76.29 ± 0.16 ^cB^	**
	**30**	88.37 ± 0.37 ^aB^	82.47 ± 0.29 ^bB^	76.45 ± 0.36 ^cB^	**
	**45**	87.59 ± 0.67 ^aC^	83.12 ± 0.21 ^bA^	76.82 ± 0.29 ^cA^	**
	**Sign.**	**	**	**	
a*	**0**	−0.29 ± 0.07 ^c^	3.41 ± 0.03 ^bA^	3.83 ± 0.03 ^aA^	**
	**15**	−0.28 ± 0.09 ^c^	3.44 ± 0.05 ^bA^	3.79 ± 0.08 ^aA^	**
	**30**	−0.27 ± 0.05 ^c^	3.17 ± 0.10 ^bB^	3.48 ± 0.17 ^aB^	**
	**45**	−0.25 ± 0.09 ^c^	2.59 ± 0.05 ^bC^	3.30 ± 0.05 ^aC^	**
	**Sign.**	ns	**	**	
b*	**0**	10.78 ± 0.12 ^cB^	12.86 ± 0.11 ^bC^	14.71 ± 0.07 ^aC^	**
	**15**	10.68 ± 0.26 ^cB^	12.89 ± 0.12 ^bBC^	14.77 ± 0.12 ^aC^	**
	**30**	10.89 ± 0.04 ^cB^	12.98 ± 0.08 ^bB^	15.13 ± 0.17 ^aB^	**
	**45**	11.16 ± 0.28 ^cA^	14.11 ± 0.06 ^bA^	16.57 ± 0.19 ^aA^	**
	**Sign.**	**	**	**	

Note: The data are presented as means ± SD. Means within a row with different letters are significantly different by Tukey’s post hoc test. Abbreviation: ns, not significant. ** Significance at *p* < 0.01. Small letters show differences among the different samples and capital letters show differences for the single sample during the storage period.

**Table 3:** Variation of the mayonnaise antioxidant parameters at 1st and 45th day of storage.

	Time (Day)	EMPE_A_	EMPE_B_	Sign.
**DPPH**	1st	78 ± 14	134 ± 11	**
	45th	74 ± 11	100 ± 1	*
	**Sign.**	**n.s.**	**	
**ABTS**	1st	463 ± 50	613 ± 74	**
	45th	590 ± 38	752 ± 146	**ns**
	**Sign.**	**	**ns**	
**TPC**	1st	323 ± 8	413 ± 18	**
	45th	353 ± 18	404 ± 28	*
	**Sign.**	*	**ns**	
**Hydroxytyrosol**	1st	82.75 ± 1.06	66.63 ± 0.18	**
	45th	44.00 ± 0.17	39.21 ± 0.08	**
	**Sign.**	**	**	
**Tyrosol**	1st	19.28 ± 0.39	18.12 ± 0.16	**ns**
	45th	12.61 ± 0.07	10.32 ± 0.04	**
	**Sign.**	**	**	
**Clorogenic Acid**	1st	4.61 ± 0.02	1.93 ± 0.06	**
	45th	3.18 ± 0.04	1.26 ± 0.01	**
	**Sign.**	**	**	
**Vanillic Acid**	1st	0.43 ± 0.02	1.66 ± 0.08	**
	45th	0.37 ± 0.02	0.34 ± 0.02	**ns**
	**Sign.**	**ns**	**	
**Caffeic Acid**	1st	2.95 ± 0.08	2.25 ± 0.06	*
	45th	2.18 ± 0.18	1.90 ± 0.08	**ns**
	**Sign.**	*	*	
***p*** **−cumaric Acid**	1st	6.50 ± 0.11	7.06 ± 0.08	*
	45th	2.55 ± 0.06	4.36 ± 0.07	**
	**Sign.**	**	**	
**Oleuropein**	1st	32.45 ± 0.20	32.55 ± 0.64	**ns**
	45th	28.74 ± 0.13	25.16 ± 0.04	**
	**Sign.**	**	**	

**Note:** The data are presented as means ± SD. Abbreviation: ns, not significant. ** Significance at *p* < 0.01; * Significance at *p* < 0.05. µmol TE 100 g^−1^ PE for ABTS and DPPH and mg kg^−1^ for TPC and single phenolics.

## Data Availability

The data presented in this study are available on request from the corresponding author.
